# Grandparenthood, Caring for Grandchildren, and Mortality in China

**DOI:** 10.1093/geroni/igaf122.1893

**Published:** 2025-12-31

**Authors:** Linghan Ge, Ming Wen, Yuying Tong

**Affiliations:** The University of Hong Kong, Hong Kong, China (People’s Republic); The University of Hong Kong, Hong Kong, China (People’s Republic); Chinese University of Hong Kong, Hong Kong, China (People’s Republic)

## Abstract

This study addresses the research gap concerning the relationship between grandparenthood, intergenerational caregiving, and mortality in non-Western contexts. Existing theories propose both benefits and drawbacks: role enhancement theory highlights the protective effects of increased emotional support, physical activity, and cognitive stimulation, whereas role strain theory points to the risks posed by excessive responsibilities, intergenerational conflicts, and energy depletion. Utilizing data from the 2011-2018 China Health and Retirement Longitudinal Study, encompassing 9,336 respondents aged 50 and above in 2011 (34,976 observations), we applied logistic regression with random effects to examine the impact of grandparenthood and caregiving patterns, including no grandchildren, having grandchildren without caregiving, and caring for grandchildren, on mortality, exploring mediation by living proximity and meeting frequency with children. Grandparenthood significantly reduces mortality risk versus having no grandchildren. Grandparents not caregiving had 26.3% lower odds of death (OR = 0.737, p = 0.041), while caregiving grandparents had 31.7% lower odds (OR = 0.683, p = 0.012). KHB mediation analysis indicated that reduced living distance and increased meeting frequency with children partly explain this effect. Living proximity mediated 24.2% (β=-0.074, p < 0.001) and 10.6% (β=-0.040, p = 0.001) of the effect for non-caregiving and caregiving grandparents, respectively; meeting frequency mediated 15.9% (β=-0.049, p = 0.001) and 5.1% (β=-0.019, p = 0.026). This research underscores the protective effects of grandparenthood and caregiving on mortality among older Chinese adults, with living proximity and meeting frequency as critical mediators, offering insights into family mechanisms promoting healthy aging in non-Western contexts.

